# Dual mobility total hip replacement: a 15-year experience in Burkina Faso

**DOI:** 10.11604/pamj.2022.41.207.27189

**Published:** 2022-03-14

**Authors:** Malick Diallo, Théodore Ouédraogo, Jean-Louis Debiesse, Jean Philippe Fayard, Paul-Henri Hulin, Joseph Millon, Wendpanga Rodrigue Lucas Douamba, Alidou Porgo, Patrick Wendpouiré Hamed Dakouré

**Affiliations:** 1 Service d’Orthopédie Traumatologie, Centre Hospitalier Universitaire Sourô Sanou, Bobo-Dioulasso, Burkina Faso,; 2 Institut Supérieur des Sciences de la Santé (INSSA), Université Nazi Boni, Bobo-Dioulasso, Burkina Faso,; 3 Service de Chirurgie, Polyclinique Notre Dame de la Paix, Ouagadougou, Burkina Faso,; 4 Unité de Formation et de Recherche en Sciences de la Santé (UFR/SDS), Université de Ouagadougou, Ouagadougou, Burkina Faso,; 5 Association Médicale du Faso, Ouagadougou, Burkina Faso,; 6 Service d´Orthopédie Traumatologie, Centre Hospitalier Universitaire de Bogodogo, Ouagadougou, Burkina Faso

**Keywords:** Total hip replacement, dual mobility, trauma sequalae, low-income country, outcome

## Abstract

To report our 15 years of experience in dual mobility total hip replacement (THR) in Burkina Faso through a Franco-Burkinabé relief organization. A retrospective study spanning from 2004 to 2018 was held in a private facility. All dual mobility THR cases with at least one year of follow-up time were included. The survey used a questionnaire, and data were analyzed with statistical software (Stata® v.13). A total of 145 primary THR in 129 patients were included in disabled young patients. There was 60.46% of males (n=78) with a mean age of 44.57 years (SD=12.43). The mean etiologies were avascular necrosis of the hip (n=84), followed by childhood chronic arthritis sequalae (n=24, 16.55%) and trauma sequalae (n=13, 8.97%). All prostheses were metal-on-polyethylene from Zimmer-Biomet®. It was usually small sizes with 48 mm (females) and 50 mm (males) cups, stem 1 (female) and 3 (males). After 2.70 years (SD=2.66) of mean follow-up times, results were good despite a high rate of revision (n=10, 6.89%) due to infections and implant malposition. THR practice might be encouraged in developing countries. The dual mobility concept is adapted to sociological activities. High duration implants and cost limitation is mandatory for the replacement joints diffusion.

## Introduction

The Bousquet concept of dual-mobility total hip replacement (THR) reduces dislocation risk [1-3]. It associates a small joint between the head and a retentive polyethylene and a large joint between the polyethylene and a metallic cup [2]. Dual-mobility THR shows long-term stability and survival [1-5]. Joint replacement data from low-income countries are paucity reported in the literature [6-8]. There is cost-limitation access to specialized care [9,10]. Also, socio-demographic features and indications are different from developed countries [7,10,11].

This study aims to report our 15 years of dual mobility THR experience through a Franco-Burkinabé relief organization, emphasizing indications, challenges, and limitations.

## Methods

**Study area:** the study setting was the surgery department of the Polyclinique Notre Dame de la Paix (PNDP). PNDP is the largest private care facility in the country. It is in Ouagadougou, the capital of Burkina Faso, West Africa.

**Study design:** this study was a retrospective longitudinal cohort study and was conducted from 2004 to 2018.

**Study population, sample size estimation, and sampling technique:** the present study included all dual mobility THR cases with at least twelve months of follow-up time. Cases with incomplete data were not included in the study.

**Study variables:** five groups of variables were studied: the sociodemographic features included patient age, sex, the hip side, hemoglobin, and other condition; injury etiology, acetabular aspect, and femoral canal constituted injury patterns; procedure aspects such as anesthesia, approach, physiotherapy, and revision were evaluated; implants characteristics (cup type and size, stem type and size, reconstruction cage device); and outcome features (follow-up, complaints, anatomic results, Harris hip score) were also assessed.

**Data collection tools:** with a semi-structured questionnaire, the survey collected the data.

**Statistical analysis:** the data were recorded on a microcomputer using the Stata version 13 (StataCorp®, College Station, Texas, USA) software with double-checking the data before data analysis. We used the Harris hip score (HHS) [12] to assess the anatomic and functional outcomes. The mean (with 95% confidence interval or with standard deviation) and the median for each quantitative variable were calculated; the results by category of the percentage of the qualitative variable were fit.

**Ethical considerations:** the study matches Helsinki's recommendations [13]. The participation to the study was determined by a free, informed, and verbal consent. Data were anonymized and patients' confidentiality was protected in accordance with the hospital ethical considerations.

## Results

**Figure 1:**
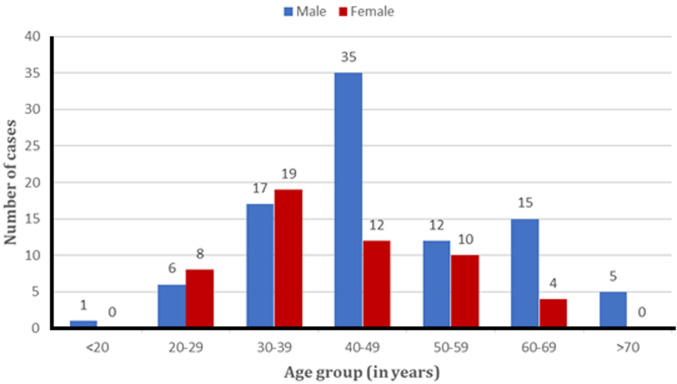
age range distribution according to sex

**Socio-demographic features:** the study involved 145 THR in 129 patients. There was 60.46% of males (n=78). The mean age at surgery time was 44.57 years (SD=12.43) range, from 19 to 77. The mean group age was between 40 and 49 years in males and between 30 and 39 years in females (Figure 1).

**Figure 2:**
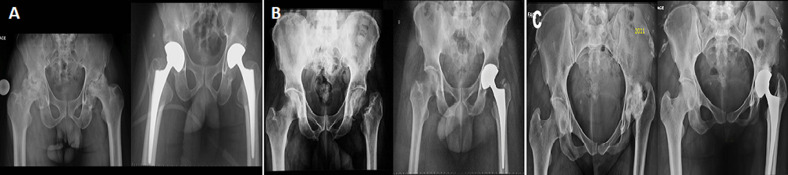
A) preoperative and postoperative AP pelvic radiographs demonstrating avascular necrosis in hemoglobinopathy; B) a femoral neck non-union; C) a childhood chronic arthritis sequalae

**Injury patterns:** we performed THR in highly disabled patients. Our patients had pain and stiff hips with an inability to walk 200 m or ride a motorcycle. At night, the pain was present at extended sit up to 30 minutes, and with weight-bearing load, requiring daily pain relief drugs. Stiffness started with a loss of rotations and range of motion limitation, to fixed adducted and flexed hips. The mean preoperative HHS was poor (42%), range from 18 to 56. Patients sustained mainly hemoglobinopathies diseased femoral head avascular necrosis (AVN) in 57.93% of cases (n=84), childhood chronic arthritis sequalae in 16.55% (n=24), and trauma sequalae in 8.97% (n=13) (Table 1). The common hemoglobinopathies were the genotypes AC (n=27) and AS (n=28). Sickle cell disease genotypes SC and SS genotypes were less present. The mean age at surgery time was more earlier in SS (mean=25, SD=5.29) and AS (mean=38, SD=8.54) genotypes and late in SC (mean=41.54, SD=13.20) and AC (mean=52.17, SD=12.60) groups. Injuries lasted from one year in trauma cases to 30 years in AVN and childhood hip chronic osteomyelitis sequalae (CHCOS) (Figure 2).

**Table 1:** repartition of THRs cases indications

Etiology	Number	Total	Percentage
Avascular necrosis		84	57.93
Hemoglobinopathy AS	28		
Hemoglobinopathy AC	27		
Sickle-cell disease SS	11		
Sickle-cell disease SC	3		
Corticoid-induced	2		
HIV induced	2		
Alcohol-induced	1		
Undetermined	10		
Childhood chronic arthritis sequalae		24	16.55
Trauma sequalae		13	8.97
Femoral neck non-union	8		
Acetabular mal union	2		
Others	3		
Primary osteoarthritis		10	
Others (congenital dysplasia, gout, rheumatism, etc.)		14	9.66
Total		145	100.00

**Procedures and implants:** procedures were planned with prosthesis layers and the BFIT® 2D software (Zimmer-Biomet®) through plain AP pelvic radiographs. We also used the HIPPLAN® 3D software (Symbios®) to assess Computed Tomography (CT)-scan, in difficult hips with the deformed acetabulum and obstructed hemoglobinopathies femurs. Patients were positioned in a lateral recumbent. Surgery was performed under spinal anesthesia through a standardized posterior approach. In fixed hips, a significant posterior muscular release was required. After surgery, patients underwent a minimum of 10 standardized physiotherapy sessions.

We used cementless and cemented titanium stem (AURA I and AURA II, Zimmer-BiometÂ®) and metal-back cementless and cemented cup (avantage and liberty, Zimmer-BiometÂ®) (Figure 2). In two cases, it was a hybrid THR with a cemented acetabular component. In other cases, it was a cementless prosthesis. The femoral stem size was usually 1 (n=20, 39.21%) in females and 3 (n=19, 21.11%) in males, ranging from 1 to 8. The acetabular cup size was 48 (n=17, 33.33%) and 50 (n=22, 24.44%) in females and males, ranging from 44 to 58. All prostheses were metal-on-polyethylene (MoP).

**Outcome:** the mean follow-up time was 2.70 years (SD=2.66) range, from one to 15 years. Four patients (4.10%) died between two and four years after surgery from intercurrent diseases. We found some anatomic complications such as hip dislocations (n=3, 2.06%), cup malposition, vertical or high (n=2, 1.37%), cup loosening (n=6, 4.13%), periprosthetic lucensis (n=4, 2.75%), calcar fractures (n=1, 0.68%) and, great trochanter (GT) stables fractures (n=3, 2.06%). It was no loosening for the femoral component. Patients complained an intermittent pain (n=2, 1.37%), a sciatic palsy (n=2, 1.37%), 15 mm to 30 mm limb discrepancy (n=6, 4.13%), a surgical site infection (n=3, 2.06%). Revision surgery was indicated in 10 cases (6.89%). We choose to perform six cup revisions, one femoral and acetabular revision after infection, one dislocation reduction, one implant removal, and one abstention. For the last two cases, no revision implant was available for revision. In the nine other cases of anatomic complications, no revision was needed. The outcome was effective in pain relief and function; it was less effective for limb lengthening because of hip injury chronicity and preoperative discrepancy in CHCOS. Mean postoperative HHS was 81.8, range from 96 to 64.

## Discussion

The current study limitations included the sample size, the annual frequency variability, the learning curve, and the multi-operator bias. Our study demonstrated a good short-term outcome in primary 145 dual-mobility THR. To our knowledge, it the most extensive survey on dual-mobility THR in a low-income country. Despite the paucity in international literature, total joint replacements (TJR) are practiced in sub-Saharan African low-income countries [7,14-18]. The annual TJR rate is a few [7,14-18]. Burkina Faso is a low-income sub-Saharan Africa country with a gross national income (GNI) of ≤1,970 $ per capita, as calculated by the World Bank Atlas method [19]. There is no national joint registry. It was around only 150 THR made in six facilities in 2019 by local surgeons and relief organizations.

The current study's mean age at surgery time was younger, 44 years. The male predominance might be explained by the more function demand and economic bias. In developed countries, THR is mainly indicated in osteoarthritis and hip dysplasia [1,5]. In Burkina Faso, we face hemoglobinopathies, especially sickle-cell disease (SCD) AVN and childhood chronic hip osteomyelitis, as hips disability primary etiology [20,21]. According to Ouedraogo *et al*. about half of hip disability is linked to hemoglobinopathy with the same mean age [20]. Hemoglobinopathy is a common condition in sub-Saharan African, affecting around 3% of the population [20-22]. It leads to early hip osteoarthritis through a femoral head AVN [23]. Counter to the statement that genotypes SS and SC gave more femoral AVN [20,23], we encounter mainly femoral head AVN in the genotype AC. This genotype gave late AVN compared to SS and AS genotypes. THR in hemoglobinopathy presents a bleeding and femoral fracture risk due to the bone condition and the narrowness of the femoral intramedullary canal [24,25].

Childhood osteomyelitis is a public health problem in low-income countries due to missed, delayed, and inadequate care [26,27]. The hip growth stops, and patients present small and stiff hips at adult age compared to the contralateral side. We encountered small sizes of implants at the femoral side and the acetabular side, even if childhood hip chronic osteomyelitis sequalae cases were excluded. Preoperative planning is mandatory in highly remained hips and to secure correct implant sizes in limited availability of implants. Our perioperative mortality was nil. The immediate postoperative period was marked by common complications such as leg discrepancies, sciatic palsies, calcar fracture, cup malposition. Learning curves and ineffective preoperative planning might explain these facts.

In the short-term, the outcome reveals intermittent pain, post-traumatic dislocations, periprosthetic lucensis, cup loosening, and surgical site infection. One dislocation was reduced under general anesthesia, and two others required acetabular revisions. Our high revision rate (6.89%) is linked to cup failures (n=6), dislocations (n=2) and infections (n=2). However, the few THR annual rates, the multi-operator condition, and the continuous learning curve explain the high revision rate. According to a prospective study from the Australian registry by de Steiger *et al*. [28], a minimum of 50 THR should be performed by a surgeon to reach the same revision rate as those performing over 100 THR. Cup failure was due to a surgical malposition and the Advantage® (Zimmer Biomet) first-generation cup coating quality, corrected in the second-generation cup. We do not experiment with any stem failure. Due to the study sample specificity such as a high hip disability in young patients, dual-mobility THR outcome was better. Common hip hyperflexion in our population activities does not increase THR dislocations rate significantly. Sub-Saharan African cultural activities that demand high hip flexion made dual-mobility THR an effective choice in that part of the world [11]. Even if there is cost-limitation access, THR development reduces the burden due to north sanitary.

## Conclusion

THR practice increases in low-income countries. Primary dual-mobility THR seems to fit with local hips specifications. There were limitations in facilities, surgeons, and implants-access. Development and diffusion of an adequate and effective prosthesis is a way to explore.

### What is known about this topic

- Total hip replacement (THR) in Africa low-income is understudied, especially dual-mobility THR;
- The main THR indication in younger in Africa is avascular necrosis (AVN) from sickle cell disease (SCD);
- Cup sizes are large in the Caucasian population.

### What this study adds

- It is the largest period and the size of the sample for a dual mobility THR in sub-Saharan Africa;
- The high rate of AVN from AS type hemoglobinopathy compared to SCD (SS or SC hemoglobinopathies);
- The cup size is small in our population.
